# Is there frequency-specificity in the motor control of walking? The putative differential role of alpha and beta oscillations

**DOI:** 10.3389/fnsys.2022.922841

**Published:** 2022-10-28

**Authors:** Charalambos C. Charalambous, Avgis Hadjipapas

**Affiliations:** ^1^Department of Basic and Clinical Sciences, Medical School, University of Nicosia, Nicosia, Cyprus; ^2^Center for Neuroscience and Integrative Brain Research (CENIBRE), Medical School, University of Nicosia, Nicosia, Cyprus

**Keywords:** stroke, walking, neural oscillations, neurorehabilitation, coherence, non-invasive brain stimulation, corticospinal tract, corticoreticulospinal tract

## Abstract

Alpha and beta oscillations have been assessed thoroughly during walking due to their potential role as proxies of the corticoreticulospinal tract (CReST) and corticospinal tract (CST), respectively. Given that damage to a descending tract after stroke can cause walking deficits, detailed knowledge of how these oscillations mechanistically contribute to walking could be utilized in strategies for post-stroke locomotor recovery. In this review, the goal was to summarize, synthesize, and discuss the existing evidence on the potential differential role of these oscillations on the motor descending drive, the effect of transcranial alternate current stimulation (tACS) on neurotypical and post-stroke walking, and to discuss remaining gaps in knowledge, future directions, and methodological considerations. Electrophysiological studies of corticomuscular, intermuscular, and intramuscular coherence during walking clearly demonstrate that beta oscillations are predominantly present in the dorsiflexors during the swing phase and may be absent post-stroke. The role of alpha oscillations, however, has not been pinpointed as clearly. We concluded that both animal and human studies should focus on the electrophysiological characterization of alpha oscillations and their potential role to the CReST. Another approach in elucidating the role of these oscillations is to modulate them and then quantify the impact on walking behavior. This is possible through tACS, whose beneficial effect on walking behavior (including boosting of beta oscillations in intramuscular coherence) has been recently demonstrated in both neurotypical adults and stroke patients. However, these studies still do not allow for specific roles of alpha and beta oscillations to be delineated because the tACS frequency used was much lower (i.e., individualized calculated gait frequency was used). Thus, we identify a main gap in the literature, which is tACS studies actually stimulating at alpha and beta frequencies during walking. Overall, we conclude that for beta oscillations there is a clear connection to descending drive in the corticospinal tract. The precise relationship between alpha oscillations and CReST remains elusive due to the gaps in the literature identified here. However, better understanding the role of alpha (and beta) oscillations in the motor control of walking can be used to progress and develop rehabilitation strategies for promoting locomotor recovery.

## Introduction

Walking is a motor task that humans practice and execute throughout their life span. It is any movement that entails displacing the center of mass of a body from one point to the other (Winter, 1991); therefore, walking is a vital motor task that when impaired can detrimentally alter the quality of life. Though it is considered to be a simple, easy, and automatic motor process, walking is a very complex task that entails the simultaneous interaction of several body organ systems with the surrounding environment (Nielsen, 2003). A crucial component of this complex interaction is the neural control of walking, which entails the interaction of the spinal, sensory, and descending input (Nielsen, 2003). The descending input, which is mainly derived from the corticospinal tract (CST) and corticoreticulospinal tract (CReST), was considered having the lesser role during walking, especially in quadrupeds (Duysens and Van de Crommert, 1998). Yet, this assumption has changed with the development of quantitative data acquisition and analysis approaches that could assess the descending drive during human walking, both neurotypical and neurologically impaired (Petersen et al., 1998; Christensen et al., 2001; Miyai et al., 2002; Suzuki et al., 2008; Barthelemy et al., 2011; Jang and Seo, 2021). Such an innovative and novel quantitative approach, which added to this evidence, is the assessment of neural oscillations during walking, both in neurotypical (Hansen et al., 2001; Halliday et al., 2003; Petersen et al., 2012; Roeder et al., 2018; Jensen et al., 2019; Weersink et al., 2021) and neurologically impaired patients (Hansen et al., 2005; Norton and Gorassini, 2006; Nielsen et al., 2008; Kitatani et al., 2016; Lodha et al., 2017; Yokoyama et al., 2020; Weersink et al., 2022; Zipser-Mohammadzada et al., 2022).

Neuronal activity across the nervous system can be characterized by oscillatory activity, whose synchronization may reflect communication across individual and populations of neurons (Buzsáki and Draguhn, 2004; Schnitzler and Gross, 2005). These local and remote networks of oscillatory activity can be distinctly classified in frequency bands (delta-δ: 1–3 Hz; theta-θ: 4–7 Hz; alpha-α: 8–13 Hz; beta-β: 14–30 Hz; gamma-γ: 30–80 Hz; fast, 80–200 Hz; ultra-fast, 200–600 Hz) (Schnitzler and Gross, 2005). It is important to note that the exact definition of these ranges varies somewhat across the literature. In humans, these oscillations can be quantified using various electrophysiological techniques, such as measuring local field potentials (LFP), electrocorticography (ECoG), magnetoencephalography (MEG), electroencephalography (EEG), and electromyography (EMG). Due to the nature of human walking, which entails close coordination between brain, spinal cord and muscles, the EEG, reflecting neuronal activity of the cerebral cortex, and the EMG, reflecting the neuronal activity of a muscle, can be employed to characterize the neural oscillations during walking, either in isolation or in combination. Data collected from both modalities are digitally processed to calculate certain time domain (e.g., cumulant density) and frequency domain (e.g., coherence) derived correlation/interdependence measures (Halliday et al., 1995). Coherence is the correlation between two signals; values of 0 and 1 denotes an absence of linear relationship and strong linear relationship between signals, respectively. Corticomuscular coherence (CMC) can be calculated when EEG and EMG are simultaneously collected (EEG_*CorticalLocationMuscle*1_–EMG_*Muscle*1_), whereas intermuscular coherence (IMC_Inter_) and intramuscular coherence (IMC_Intra_) can be calculated when EMG signal from two different muscles (EMG_*Muscle*1_–EMG_*Muscle*2_) or within a muscle (EMG_*Muscle*1*A*_–EMG_*Muscle*1*B*_) is collected, respectively.

**FIGURE 1:**
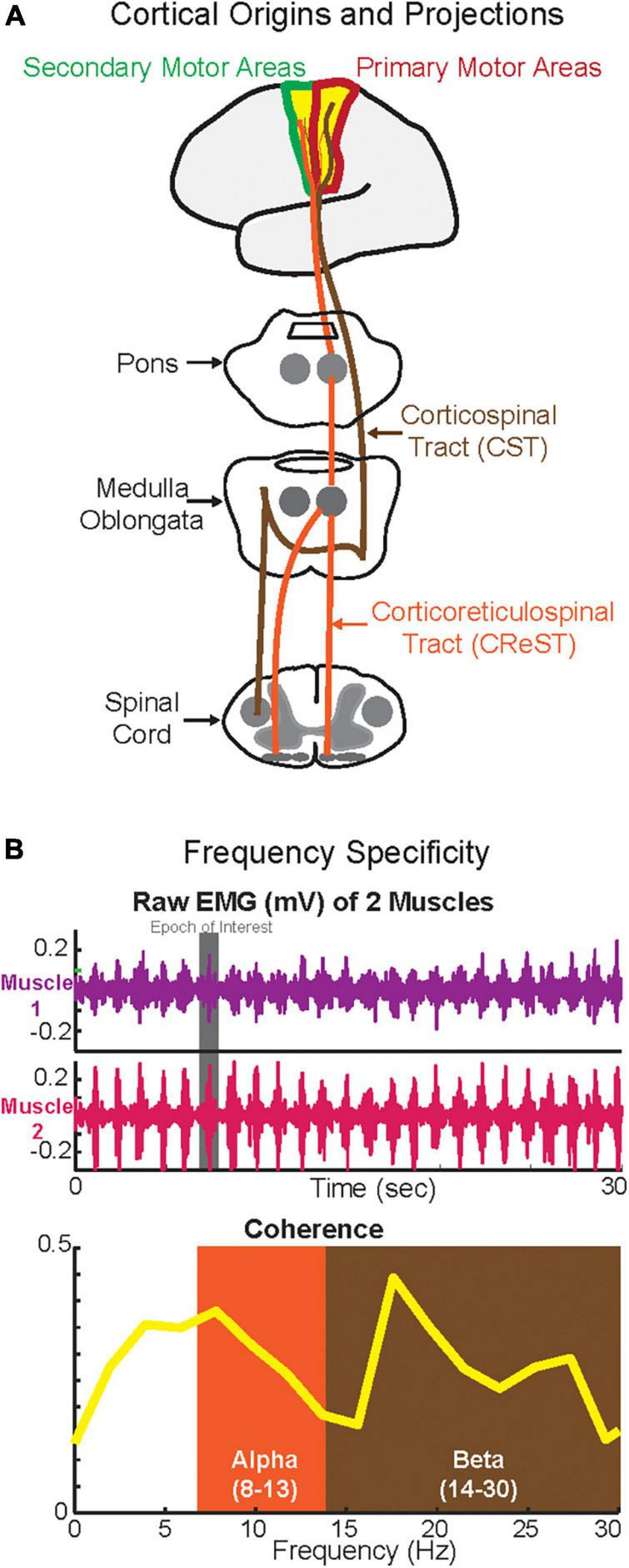
Cortical origins and the projections of corticospinal tract (CST) and corticoreticulospinal tract (CReST) **(A)** and frequency specificity **(B)**. **(A)** This is a simplified diagram of the origins and projections of the CST and CReST from the cortex to the spinal cord. **(B)** Top panel depicts phasic activity of two synergistic muscles (e.g., plantarflexors) during walking while the bottom panel depicts the coherence in alpha (putatively CReST-derived) and beta (putatively CST-derived) bands of these two muscles.

Among all oscillations, the two that have been mainly reported to be relevant during human walking are the alpha and beta oscillations, which in turn have been linked to the two major descending motor drives, CST and CReST (Figure 1A). The CST is a pathway, which is mainly monosynaptic and thus fast, and responsible for contralateral activation of distal muscles during fine and dexterous movements (York, 1987; Nathan et al., 1990; Lemon, 2008). CReST is a predominantly slow, oligosynaptic pathway and responsible for bilateral activation of axial and proximal muscles during gross movements (Peterson et al., 1979; Peterson B., 1979; Peterson B. W., 1979; Jang and Lee, 2019). Both pathways are mainly originated from the primary and secondary motor areas and to a lesser extent from the sensory areas. Yet, the primary locus of origin differs between the two tracts. Most of the CST projections are derived from the primary motor areas (Witham et al., 2016) whereas the most of CReST projections are derived from secondary motor areas (Fregosi et al., 2017). Hence, stimulation of the primary motor areas elicits mainly contralateral responses, whereas stimulation of the secondary motor areas elicits mainly ipsilateral and bilateral responses (Montgomery et al., 2013). Furthermore, in terms of force control, CST is responsible for the fine control of force, whereas CReST plays a role in monotonic increase of force (Glover and Baker, 2022). Though CReST has been always proposed to have a dominant role in walking, in the last few decades the CST has been suggested to have some role in human walking. Therefore, existing evidence suggests that both motor descending tracts play a role in walking (Nielsen, 2002; Matsuyama et al., 2004; Barthelemy et al., 2011; Kwon et al., 2017; Yeo et al., 2020; Jang and Cho, 2022).

Several studies have suggested that the motor descending drive is *frequency-specific*. Hence, the two major descending drives from CST and CREST may in fact operate at different frequencies (Figure 1B). Previous work suggested that the alpha-band oscillations quantified by IMC_Inter_ may be CReST originated; therefore, alpha oscillations can be used as a CReST proxy (Grosse and Brown, 2003). Conversely, previous work suggested that beta-band oscillations quantified by CMC and IMC_Inter_ may be CST originated; therefore, beta-band oscillations can potentially be used as a CST surrogate (Conway et al., 1995; Fisher et al., 2012). Given the feasibility of quantifying neural oscillations during walking using EEG and EMG (but see Section “Methodological considerations”), the assessment of the activity in these bands using CMC, IMC_Inter_, and IMC_Intra_ may help to further delineate the putative differential role that they have on the descending drive (i.e., frequency specificity). This is especially important in clinical cohorts with neurological disease (e.g., stroke) where a descending tract is damaged, because knowing the underlying mechanisms of these oscillations (Thibaut et al., 2017) relative to the tract that they may represent will allow for effective rehabilitation strategies to promote locomotor recovery (Jang and Kwon, 2016; Jang and Lee, 2017; Seo and Sh, 2020).

Depending on the damage to either tract, walking can be impaired (Yoo et al., 2014). Neurorehabilitation modalities that have been used to alter walking behavior in both neurologically intact and impaired cohorts are known as non-invasive brain stimulation (NIBS) techniques. These techniques are classified into two major categories: magnetic and electric stimulation (Liew et al., 2014). Most studies used either repetitive TMS (rTMS: application of repetitive magnetic pulses grouped in frequency-specific bursts to induce action potentials) or transcranial direct current stimulation (tDCS: application of low amplitude current that can modulate the threshold for discharge of cortical neurons). rTMS is used primarily offline (i.e., not during walking), whereas tDCS can be used either offline or online (i.e., during walking). Both rTMS and tDCS studies showed relatively small effects on walking behavior (Li Y. et al., 2018; Kindred et al., 2019, 2020; Tien et al., 2020; Fan et al., 2021). In addition to the methodological variability across studies and that the stimulation parameters of either NIBS tool may not be yet optimized for walking-specific stimulation, a major explanatory factor for the small effects may be that neither rTMS nor tDCS were applied to target walking-specific neural oscillations. Of these two NIBS techniques, only rTMS has the capacity to synchronize and modulate the endogenous neural oscillations in the brain areas of interest (i.e., entrainment) (Bergmann and Hartwigsen, 2021). Despite its promising use in stroke rehabilitation (Fisicaro et al., 2019), the main limitation of rTMS in this setting is that it cannot be used in real time (“online”) during walking due to its technical/instrumentation characteristics (e.g., size of the machine, consistent and precise coil stabilization over the subject’s head during walking, etc.).

Transcranial alternate current stimulation (tACS) is an alternative NIBS technique that is potentially capable of synchronizing and modifying neural oscillations online (i.e., during walking) due to more favorable instrumentation features. Applying frequency-specific tACS over the motor area of interest can modulate the targeted band, which can in turn, potentially alter the corresponding behavior *via* periodic stimulation (Vosskuhl et al., 2018). This effect of tACS on the neural oscillations has been recently attributed to two proposed mechanisms: entrainment of intrinsic oscillations and spike-timing dependent plasticity (Vogeti et al., 2022). Entrainment suggests that oscillations are synchronized by the external tACS stimulus preferentially when the stimulated frequency is close to the intrinsic frequency of the network. Plasticity, on the other hand, suggests that the tACS stimulus brings about synaptic changes based on the timing of firing in the network. Namely, synaptic connections are strengthened/weakened if pre-synaptic events occur before/after post-synaptic events, respectively. It is yet not clear whether the two proposed mechanisms are mutually exclusive or operate jointly. “Online” (i.e., real time) effects of stimulation are attributed to entrainment, whereas “offline” aftereffects effects may be better accounted for by plasticity. Despite a substantial and growing body of literature on tACS, it has only recently been applied to human walking. Despite promising results, tACS has so far only been applied in limited settings: the cortical origins of the ankle dorsiflexors were primarily targeted and mainly the beta-band was analyzed and reported (Kitatani et al., 2020a,b). Therefore, tACS could possibly alter walking by targeting specific frequency bands (Storch et al., 2021), yet this remains to be confirmed.

Having the capacity to feasibly and reliably assess the descending drive that is responsible for human walking could aid in characterizing the residual walking capacity of a stroke patient and in targeting the relevant descending drive to promote walking recovery after a neurological disease. Neural oscillations in the alpha and beta-bands during human walking may provide this capacity; however, the exact role of these oscillations in the underlying mechanisms of walking is still unclear. Hence, the remaining unanswered question is whether there is a differential role of alpha-band and beta-band oscillations in the motor descending drive (CReST vs. CST) both in neurotypical and neurologically impaired walking in humans.

In this review, we aim to present and discuss the existing evidence on the differential role of the alpha- and beta-band oscillations during human walking; thus, to examine whether the motor control during walking may be frequency-specific (Alpha/CReST vs. Beta/CST). First, we will synthesize and discuss the existing evidence on whether alpha-band and beta-band oscillations can be viewed as proxies of CReST and CST, respectively. Second, we will examine the role of each band during neurotypical and neurologically impaired (i.e., stroke) walking, and how external stimulation (i.e., tACS) can modulate these bands and subsequently change walking behavior. Finally, we will present the remaining gaps in knowledge, future directions, and methodological considerations related to the data acquisition, extraction, and analysis of walking-related neural oscillations. This evidence will be mainly retrieved from studies that used EEG and EMG, either in isolation or combination during walking, and calculated either CMC, IMC_Inter_, or IMC_*Intra*_.

## Oscillations of different frequency as proxies of the descending drive

In recent years, neural oscillations have received great amount of attention in neurorehabilitation studies. This is because of the potential use of each frequency band as a biomarker/surrogate of each motor descending pathway (see Figure 1). Given their role during walking, the structural and functional characterization of both CReST and CST is important, especially after damage to one of the tracts due to neurological disease (e.g., stroke). Though there are several functional and structural methodological tools to characterize both tracts, only a few can feasibly characterize them during dynamic tasks, such as walking. The relative feasibility of collecting EEG and EMG and calculating cumulant densities and coherences during walking makes the quantification of oscillations at in different frequency bands an attractive approach.

In humans, CReST can be probed using various approaches, such as the startle acoustic response (SAR) (Rothwell, 2006; Valls-Sole et al., 2008) and TMS (Ziemann et al., 1999; Maitland and Baker, 2021; Taga et al., 2021). Briefly, the former (i.e., a response to an unexpected acoustic stimulus) has been shown to activate the reticular formation of the lower brain stem and to subsequently activate the reticulospinal efferent of CReST (Yeomans and Frankland, 1995). Conversely, TMS has been suggested to activate indirectly the pontomedullary reticular formation by applying high intensity stimulus while the ipsilateral target muscle is contracted at a percentage of maximum contraction (Ziemann et al., 1999; Taga et al., 2021). Using SAR, Grosse and Brown (2003) recruited healthy adults who were asked to slightly contract several proximal and distal upper extremity muscles while (1) unexpected acoustic stimuli were delivered (i.e., startle condition), (2) a startle response was mimicked (i.e., sham startle condition), or (3) a subgroup of the target muscles were tonically contracted bilaterally (i.e., voluntary contraction condition). EMG from all target muscles were used to calculate the IMC_inter_ of homologous muscles. The main findings were that the proximal muscles (i.e., deltoid and biceps) had significant coherence between 10 and 20 Hz with discrete peaks around 12–14 Hz only in the startle condition, whereas the distal muscle (i.e., first dorsal interosseous) showed no significant coherence in any condition. These findings suggested that this common oscillatory activity at 12–14 Hz in the homologous deltoid and biceps muscles is due to reticulospinal efferent drive of CReST. This postulation was based on three findings. First, the oscillatory activity in this band was elicited only in the startle condition (i.e., SAR activates reticular formation). Second, this oscillatory activity was present only in the proximal muscles, whose descending drive is mainly from CReST, but not in the distal, whose descending drive is mainly from CST. Third, the significant coherences were bilateral (i.e., consistent with the typical CReST innervation) whereas coherences in the distal muscles were unilateral (i.e., consistent with typical CST innervation). Although these findings strongly suggest that 10–20 Hz band may be a potential proxy of CReST activity, we note that this range is not the typical range for quantifying the alpha band (i.e., 8–13 Hz). Based on the conventional classification of frequency ranges, the range used here was a combination of high alpha and low beta band. Yet, the results from this study showed discrete peaks around 12–14 Hz. Therefore, regardless of this methodological consideration, these findings paved the way for the notion that frequency-specific oscillations in the intermediate frequency range may be a surrogate of CReST.

Conversely, numerous studies have suggested beta-oscillations as a potential surrogate of CST. This notion is based on various sources of evidence. First, several studies that used MEG and EMG demonstrated strong synchronization in the beta-band (16–32 Hz) between motor cortex and contralateral muscle (Conway et al., 1995; Salenius et al., 1997; Kilner et al., 2000); CST innervates mainly the contralateral muscles (Davidoff, 1990; Nathan et al., 1990). Second, it is present in neurotypical adults and in patients with progressive muscular atrophy (both groups have intact CST), but it is absent in patients with primary lateral sclerosis (CST is impaired) (Fisher et al., 2012). Therefore, the presence of beta-band oscillations is dependent on CST integrity. Third, beta oscillations originate from the anterior bank of the central sulcus (i.e., primary motor cortex) and have somatotopic properties (medial fissure for lower extremities and lateral to medial fissure for upper extremities) (Salenius et al., 1997). Similar somatotopic property exists for CST as well (Alkadhi et al., 2002; Conti et al., 2014). Fourth, a recent study showed that the beta-oscillations are most likely carried only by the fast conducting CST axons (i.e., monosynaptic connections *via* corticomotoneuronal axons) (Ibanez et al., 2021). Taken together, all these four pieces of evidence suggest that beta-band can be used as a surrogate for CST, at least for the fast-conducting axons (Ibanez et al., 2021).

In addition to the aforementioned empirical evidence, from a theoretical stand point this frequency specificity may be explained by the anatomical and physiological characteristics of each tract. Given that CST is a fast-monosynaptic tract with contralateral direct projections (i.e., direct communication from cortex to the contralateral alpha-motoneurons in the spinal cord–see Figure 1A), the time delay of the descending drive should be short; therefore, in the setting of continuous oscillatory activity, one could expect this shorter delay would translate to higher frequencies. Conversely, given that CReST is a slow oligosynaptic tract with bilateral indirect projections (i.e., from cortex to pons and medulla and then to bilateral interneurons in the spinal cord–see Figure 1A), the time delay of the descending drive should be long; therefore, in the presence of ongoing oscillations, the frequency may be expected to be lower as in the case of CST. Yet, such postulations remain merely theoretical and must be empirically tested in animal models. This represents an important gap in the literature.

## Characterization of alpha and beta band during neurotypical walking

**FIGURE 2:**
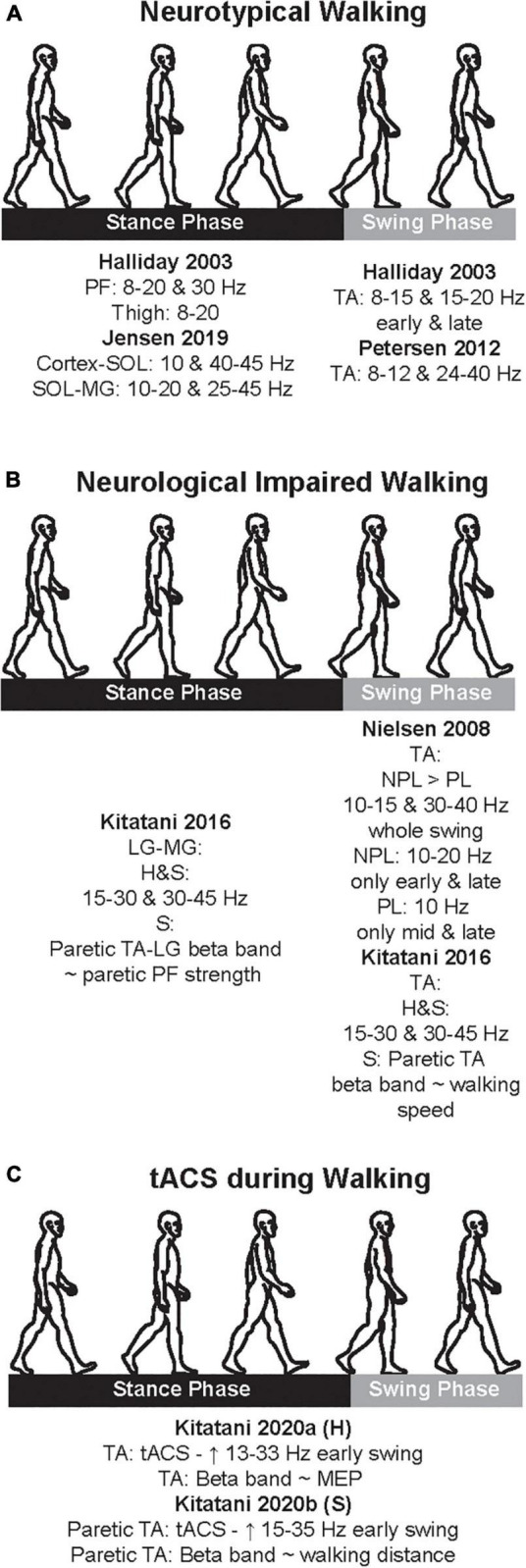
Cumulative summary of the main findings of the studies that examined coherences during neurotypical walking **(A)**, neurological impaired walking **(B)**, and transcranial alternate current stimulation (tACS) application **(C)**. Here, we just present the main findings; see text for further details. PF, plantarflexors; TA, tibialis anterior; LG, lateral gastrocnemius; MG, medial gastrocnemius; H, healthy; S, stroke; NPL, non-paretic leg; PL, paretic leg; ∼, significant correlation; MEP, motor evoked potential.

Since the early 2000s, there have been numerous studies, which characterize neural oscillations during walking in both neurotypical and clinical cohorts. In this and next section, we will present and discuss the studies which examined the neural oscillations during neurotypical walking (this section) and neurologically impaired walking after stroke (next section) with special emphasis on studies whose findings can contribute to the main question of this review (i.e., is there frequency-specificity in the motor control of walking?). To quantify the oscillations, early studies used simpler methodological approaches, whereas later studies applied more complex data analyses. Also, a few studies did not explicitly define the frequency bands, they just reported the ranges of significant results. Furthermore, studies had neurotypical adults and stroke patients walk on a treadmill or over ground and focused on specific muscle groups that have a functional role during walking and specific descending input. Table 1 summarizes the methodological characteristics of the studies, which examined alpha and beta bands during neurotypical walking, while Figure 2A depicts a cumulative summary of the main findings reported in those studies in neurotypical adults.

**TABLE 1:** Characteristics of studies that examined oscillations during neurotypical walking.

		Data acquisition				Data analysis		
Study	Sample size	Testing modality	Walking speed (m/s)	Muscles	EEG and/or EMG	Gait cycle phase	Frequency domain measures	Frequency domain bands (Hz)
Hansen et al., 2001	25 6 15	TMW TMW Tonic DF	0.83–1.11 0.22–1.66 N/A	TA, SOL, MG, LG, BF, VL, VM	EMG	All muscles: whole	IMC_Intra_: TA IMC_Inter_: rest of the muscles	N/A
Halliday et al., 2003	10	TMW	1.11	TA, SOL, MG, LG, BF, VL, VM	EMG	TA: swing Rest of muscles: stance	IMC_Intra_: TA IMC_Inter_: rest of the muscles	5–50
Gwin et al., 2011	8	TMW TMR Stance	0.8–1.25 1.9 N/A	N/A	EEG	N/A	Power	Alpha: 8–12 Beta: 13–30 Gamma: 50–150
Petersen et al., 2012	9	TMW TMW Tonic DF	0.27 0.97–1.11 N/A	TA	EEG EMG	Swing	CMC	8–12 24–40
Jensen et al., 2019	11	TMW	1	SOL, MG	EEG EMG	Push off during stance	CMC IMC_Inter_	5–50

TMW, treadmill walking; TMR, treadmill running; DF, dorsiflexion; N/A, not applicable; TA, tibialis anterior; SOL, soleus; MG, medial gastrocnemius; LG, lateral gastrocnemius; BF, biceps femoris; VL, vastus lateralis; VM, vastus medialis; EEG, electroencephalography; EMG, electromyography; IMC_Intra_, intra-muscular coherence; IMC_Inter_, inter-muscular coherence; CMC, corticomuscular coherence.

Early attempts for elucidating the common descending drive to lower limb muscles during treadmill walking examined the synchronization of motor unit activity in neurotypical adults. Motor unit discharges can be synchronized either short-term or long-term (Hansen et al., 2001). The short-term synchronization has a short duration (average of ∼12 ms) and high amplitude and depends on pyramidal tract activity. Conversely, the long-term synchronization has longer duration and lower amplitude. Hansen et al. (2001) examined this type of synchronization in neurotypical adults who walked at a comfortable speed and variable speed range (i.e., to test the effect of speed on the synchronization) and tonically dorsiflexed their ankle (i.e., to compare the synchronization between walking and tonic dorsiflexion). Synchronization using coherence was quantified for tibialis anterior (TA; IMC_Intra_) using both wire and surface EMG recordings, however, for soleus (SOL), medial and lateral gastrocnemius (MG and LG), biceps femoris (BF), vastus lateralis (VL), and vastus medialis (VM) only surface EMG recordings were used. The main finding was that there was a short-term synchronization within (IMC_Intra_) and between (IMC_Inter_) muscles during walking as it was reported in tonic contraction. Specifically, the strongest short-term synchronization was recorded in TA and was present predominantly during early and late swing. Also, in some subjects, there was a short-term synchronization for synergistic muscles (e.g., plantarflexors: SOL, MG, LG), which shared the same action around a common joint (e.g., ankle). There was no effect of walking speed on the synchronization measures. Using coherence analyses, these findings suggested the potential role of the pyramidal tract’s activity to the lower limb muscle activity during walking, especially for the TA and to a lesser extent for the ankle and knee synergistic muscles. Though this study did not explicitly report any oscillations and examined EMG activity throughout the gait cycle, they paved the path to subsequent studies to examine the common drive to lower limb muscles during walking.

Halliday et al. (2003) assessed and reported specific time- and frequency-domain measures during walking in neurotypical adults, who walked at a fixed speed. Cumulant densities and coherence were analyzed for a number of lower limb muscles during specific gait phases in which the target muscles were predominantly activated (i.e., TA–swing phase; ankle plantarflexors and thigh muscles–stance phase). For the TA IMC_Intra_, significant components of the 8–15 and 15–20 Hz frequency bands were present in early and late swing, yet suppressed during mid-swing. Therefore, the common drive to the motoneuronal pool of TA can be modulated during the swing phase of gait, a period that TA is mainly activated to swing the leg forward by clearing the foot off the floor (Winter and Yack, 1987; Winter, 1992). Like TA, the IMC_Inter_ of plantarflexors during stance was observed between 8 and 20 Hz and close to 30 Hz, yet its magnitude was lower than TA IMC_Intra_. Thigh muscles IMC_Inter_ during stance was also observed between 8 and 20 Hz; however, its size was lower than the IMC_Intra_ of ankle plantarflexors. Like the findings from the Hansen et al. (2001), this study showed that the TA had the strongest coherences during early and late swing and that synergistic muscles around ankle and knee had significant coherences during stance. Coherences were not significant for not synergistic muscles.

The combined findings of both studies added new evidence regarding the neural control of lower limb muscles during walking. First, they pointed out that the common neural drive, which they postulated was most likely CST-derived, was the strongest for the TA, a muscle with strong CST excitability, during subphases of the swing, a gait phase that TA is predominantly active (Winter and Yack, 1987). This finding agrees with findings from TMS studies that showed modulation of TA during walking (Schubert et al., 1998; Capaday et al., 1999; Christensen et al., 2001), and also with the notion that the TA is the lower limb muscle with the strongest direct corticomotoneural connections (Brouwer and Ashby, 1992; Brouwer and Qiao, 1995; Bawa et al., 2002). Second, as TMS studies showed, the descending drive to ankle plantarflexors and thigh muscles is still present during walking but is relatively lesser than for TA (Schubert et al., 1997; Capaday et al., 1999; Iglesias et al., 2012). This adds to the notion that for these muscles the sensory input (e.g., proprioception) during walking may have greater role than the descending drive, especially from CST (Capaday et al., 1999; Sinkjaer et al., 2000; Capaday, 2002). Third, the methodological approach by Halliday et al. (2003) to analyze within and between muscles coherences only during gait phases in which the muscles of interest are active is very important. These seminal studies yielded important insights, however, what was missing was the cortical component of the common neural drive as both studies only examined the neural oscillations derived from EMG data.

Gwin et al. (2011) collected EEG signals during walking in neurotypical adults. Using high-density EEG, they estimated bilaterally the inter-stride spectral power of several frequency bands. To quantify body dynamics across gait cycles, they also collected and analyzed kinematic and kinetic data. Both datasets were collected while neurotypical adults stood, walked and ran on instrumented treadmill. No EMG of the lower limb muscles was collected; therefore, only the EEG power in each frequency band was reported by generating spectrograms for each electrocortical source across gait cycles for each participant. They showed that increase in oscillatory activity estimated to originate from/localize to the brain areas of interest (anterior cingulate, prefrontal cortex, posterior parietal cortex, and sensorimotor cortex) varied across bands and was dependent on specific gait events. Specifically, there were significant increases in alpha and beta bands around the sensorimotor and dorsal anterior cingulate cortex while the leading leg was contacting the ground and the trailing leg was pushing off the ground. Interestingly, these increases were more pronounced during contralateral than during ipsilateral push off. Beta oscillations occurred 8% of the gait cycle earlier than the alpha band. Conversely, the high gamma band had power increases in all brain areas of interest and had more peaks per gait cycle than the other two frequency bands. This study demonstrated that there are online cortical contributions to neurotypical steady state walking, and that these contributions are derived from multiple brain areas and are modulated by the gait events, especially on the contralateral side.

Though the aforementioned studies provided significant evidence about the neural oscillations during walking, collecting simultaneously EEG and EMG signals can characterize the synchrony between the two signals responsible for a muscle. Therefore, two studies from the same group combined EEG with EMG in order to investigate the synchrony between motor cortex and the ankle plantarflexors (SOL, MG, LG) and dorsiflexors (TA) during treadmill walking in neurotypical adults. The first study focused on the CMC and IMC_Intra_ of TA while the second study focused on the CMC and IMC_*Inter*_ of SOL and MG.

In the Petersen et al. (2012) study, subjects walked on their preferred and slow speed while EEG and EMG from the TA was collected. CMC of two frequency bands were analyzed, and CMC peaks (coupling) within each range were reported. During normal walking, coupling between EEG and TA EMG was present during the swing phase in 7/9 and 9/9 subjects for the 8–12 Hz and 24–40 Hz bands, respectively. Similarly, during slow walking, coupling between EEG and TA EMG was present during the swing phase in 6/9 and 7/9 subjects for the 8–12 and 24–40 Hz bands, respectively. For both walking speeds, the CMC peaks observed in 8–12 Hz were smaller than in 24–40 Hz band. During static dorsiflexion, coupling between EEG and TA EMG was present in 7/9 subjects for the 15–30 Hz band. Given that the most pronounced CMC peaks were present primarily within the 24–40 Hz band in most subjects (see normal walking) during the swing phase, the findings from this study suggested that this common neural drive to the TA most likely originates from CST. Previous studies that examined the TA IMC_Intra_ (15–50 Hz) in clinical cohorts whose CST was damaged, reported significant reductions in this band (Hansen et al., 2005; Nielsen et al., 2008; Barthelemy et al., 2010; Zipser-Mohammadzada et al., 2022). Taken together, these findings added new evidence in support of the role of the CST drive from the motor cortex (EEG) to the motor units of TA (EMG) during the swing phase of walking.

Up until the late 2010s, most studies predominantly focused on the neural oscillations, especially in the beta band, of the TA during the swing phase of walking. This focus of attention was due to the fact that beta band may be a CST proxy (Fisher et al., 2012), TA activation is mainly CST driven during walking (Capaday et al., 1999), and that TA is mainly activated during the swing phase (Winter and Yack, 1987). Jensen et al. (2019) first examined neural oscillations related to activation of ankle plantarflexors during walking. Using both EEG and EMG, they calculated the CMC and IMC_Inter_ of the ankle plantarflexors (MG and SOL) during the push off phase of stance while neurotypical adults walked on a treadmill. For the motor cortex-SOL coupling, there were significant CMC around 10 and 40–45 Hz, whereas for the SOL-MG coupling there were significant IMC_Inter_ at 10–20 and 25–45 Hz. Compared to Petersen et al. (2012), which focused on the common neural drive to TA IMC_Intra_, in this study, the frequency range was relatively greater while the magnitude of the coherences was lower. This indicates that the neural oscillations for the ankle plantarflexors are present in wide range of frequencies whereas the strength may not be as pronounced as in TA. Authors argued that the activation of the ankle plantarflexors during the stance phase of walking is also driven by CST input, which, however, may be lesser than in the case of TA due to the sensory input to this muscle group during stance phase (i.e., contact with the ground) (Sinkjaer et al., 2000; Af Klint et al., 2010).

Taken together the findings from both studies, demonstrated that both ankle plantarflexors and dorsiflexors receive input from CST during the phases of walking in which these muscles are predominantly active. As anticipated, this input seems to be greater for the ankle dorsiflexors than for the plantarflexors; consistent findings were reported in TMS work (Capaday et al., 1999). Furthermore, both studies reported peaks in 8–13 Hz band (i.e., alpha band) for both muscle groups; however, whether the origin of these peaks was related to CReST was not explicitly discussed.

## Characterization of alpha and beta band during neurological impaired walking

One of the main motor limitations after stroke is walking, and a majority experience lasting walking deficit without a recovery (Jorgensen et al., 1999). After stroke, CST is one of the descending pathways that is usually damaged and is closely related to motor impairment (Byblow et al., 2015), and both ankle plantarflexors and dorsiflexors are weakened (Li S. et al., 2018). Due to these stroke-induced damages, drop foot is observed; therefore, stroke patients have difficulty to adequately produce propulsive impulse during the push off in stance phase (i.e., function of the plantarflexors) (Bowden et al., 2006; Turns et al., 2007) and to efficiently clear the foot off the ground during the swing phase (i.e., function of the dorsiflexors) (Roche et al., 2015). Accordingly, the few studies that examined the neural oscillations in stroke patients during walking focused predominantly on these two muscles groups. Table 2 summarizes the methodological characteristics of these two studies, which examined alpha and beta bands during neurological impaired walking, while Figure 2B depicts a cumulative summary of the main findings reported in these studies.

**TABLE 2:** Characteristics of studies that examined oscillations during neurological impaired walking.

		Data acquisition				Data analysis		
Study	Sample size	Testing modality	Walking speed (m/s)	Muscles	EEG and/or EMG	Gait cycle phase	Frequency domain measures	Frequency domain bands (Hz)
Nielsen et al., 2008	8 stroke	TMW	0.11–0.28	TA	EMG	Swing	IMC_Intra_: TA	N/A
Kitatani et al., 2016	9 healthy 11 stroke	OGW	0.55 ± 0.04 0.58 ± 0.21	TA, MG, LG	EMG	TA: swing TA, MG, LG: stance	IMC_Intra_: TA IMC_Inter_: all muscles	Beta: 15–30 Gamma: 30–45

TMW, treadmill walking; OGW, over ground walking; TA, tibialis anterior; MG, medial gastrocnemius; LG, lateral gastrocnemius; EEG, electroencephalography; EMG, electromyography; IMC_Intra_, intra-muscular coherence; IMC_Inter_, inter-muscular coherence.

Nielsen et al. (2008) bilaterally examined the TA IMC_Intra_ during walking in chronic stroke patients. Analyses included the whole swing phase and the subphases of the swing (early, mid, and late swing). During the swing phase as a whole, the non-paretic leg had larger coherences (10–15 Hz–30–40 Hz) and narrower central peak synchronization than the paretic leg. When the swing phase was divided into early, mid, and late swing, a significant 10–20 Hz coherence was present in the non-paretic leg during the early and late swing; this band was not present at the mid swing. Furthermore, on the paretic side, 10 Hz peak of coherence was predominantly observed only in mid and late swing and not in early swing. This was the first evidence that TA coherence remains intact on the non-paretic side during the same swing phases that have been reported in neurotypical adults (Hansen et al., 2001; Halliday et al., 2003). Conversely, this coherence is diminished on the paretic TA and only a peak of 10 Hz is present. Given that CST is usually damaged after stroke and the fact that frequencies over 10 Hz were not visible on the paretic side, this finding adds to the notion that CST integrity is a prerequisite for the presence of beta band activity. For the 10 Hz peak on the paretic side, authors argued that it may be due to sensory input. Yet, one could also argue that this peak may be a manifestation of CReST upregulation, which has been suggested to be present after stroke (Chen et al., 2018; Jang and Lee, 2019; Jang and Cho, 2022). Whether this holds true is remains to be elucidated in future work.

Ankle plantarflexors are also impaired after stroke (Li S. et al., 2018), which contributes to walking deficits (Bowden et al., 2006; Turns et al., 2007; Brough et al., 2019). In neurotypical walking, there is no neural coupling between antagonist muscles (Hansen et al., 2001; Halliday et al., 2003). However, previous work has shown that after spinal cord injury (SCI) there was a significant coherence between quadriceps, which are hip flexors and knee extensors, and hamstrings, which are hip extensors and knee flexors, during the stance phase of walking (Norton and Gorassini, 2006). Given that such a coupling between antagonist muscles is uncommon in neurotypical walking, this coupling seems to occur after CST injury in order to facilitate functional walking.

Based on this premise, Kitatani et al. (2016) examined the coherences (i.e., 15–30 and 30–45 Hz) of both ankle plantarflexors (MG and LG) and dorsiflexors (TA) in neurotypical adults and chronic stroke patients during over ground walking. The IMC_Inter_ (LG-MG, TA-LG) and IMC_Intra_ (TA-TA) were analyzed during specific subphases of the stance and swing phase, respectively. In addition to coherences, they collected basic walking and clinical measures. For both LG-MG and TA-TA, significant coherences from beta (15–30 Hz) to low gamma (30–45 Hz) were observed in all neurotypical adults (right side) and stroke patients (non-paretic side). On the paretic side, significant coherences from beta (15–30 Hz) to low gamma (30–45 Hz) were observed for the TA-TA and LG-MG only in 10 and 8 patients, respectively. The antagonist coupling (i.e., TA-LG coherence) was present only in a single neurotypical adult and had low magnitude. Conversely, TA-LG coherence was present in stroke patients (eight non-paretic and seven paretic). Overall, the TA-TA and LG-MG coherences were greater in frequencies around beta to low gamma on the right (neurotypical adults) and non-paretic side (stroke) compared to the paretic side (stroke). Compared to neurotypical adults, the TA-LG coherence was greater in frequencies around beta to low gamma in stroke. Furthermore, the only significant correlations of coherence with behavioral measures were between TA-TA beta band of the paretic side and walking speed and TA-LG beta band of the paretic side and paretic plantarflexors strength. The former was positive (i.e., the greater the coherence within paretic TA, the faster the walking speed), while the latter was negative (i.e., the greater the antagonistic coupling around the paretic ankle, the weaker the paretic plantarflexors). Taking these findings together, the common neural drive to synergistic muscles depends on CST integrity, whereas any damage to CST due to stroke unmasks the antagonist coupling which is uncommon under neurotypical conditions. Given that this manifestation is observed also after SCI (Hansen et al., 2005), patients with CST damage may be capable of functional walking *via* the bilateral unmasking of this antagonist coupling. Rehabilitation strategies that can reverse this uncommon coupling either directly or indirectly (i.e., strengthen ankle plantarflexors) may convert the functional walking to neurotypical walking; however, whether this holds true remains to be investigated.

## Neuromodulation of alpha and beta band during walking

The studies in the previous section suggest an association between neural oscillations and walking. The causality of this association may be tested when experimentally well-controlled changes in neural oscillation are indeed shown to cause changes in walking. A NIBS tool that can induce specific brain oscillations extrinsically (and hence in a well-controlled way) is tACS, which depends on external application of oscillatory electrical activity (Antal and Paulus, 2013). Compared to other NIBS modalities that can modulate system’s overall polarity, tACS can modulate the membrane potential of the targeted region; therefore, it may have a direct effect on overall level of depolarization and hyperpolarization (Voroslakos et al., 2018). Given its ability to modulate frequency-specific oscillations, tACS can also be a powerful tool for clinical applications (Riddle and Frohlich, 2021). It is also relatively feasible to be applied in dynamic motor tasks, such as walking, due to its size and the relative ease to be carried out. Yet, surprisingly, there is limited evidence on the application of tACS during walking. To the best of our knowledge, only two studies, both conducted by the same group, investigated the effect of applying tACS over the cortex during walking. One study recruited neurotypical adults (Kitatani et al., 2020a) while the other recruited chronic patients (Kitatani et al., 2020b). Table 3 summarizes the methodological characteristics of these two studies, which examined alpha and beta bands during the application of tACS in both neurotypical adults and stroke patients, while Figure 2C depicts a cumulative summary of the main findings reported in those studies.

**TABLE 3:** Characteristics of studies that examined neuromodulation using transcranial alternate current stimulation (tACS) during both neurotypical and neurological impaired walking.

		Data acquisition				Data analysis		
Study	Sample size	Testing modality	Walking speed (m/s)	Muscles	EEG and/or EMG	Gait cycle phase	Frequency domain measures	Frequency domain bands (Hz)
Kitatani et al., 2020a	14 healthy	TMW	N/A	TA, MG, LG, VM, VL BF, ST	EMG	TA: early swing MG and LG: mid-to-terminal stance VM, VL, ST, & BF: late swing to early stance	IMC_Intra_: TA	Beta: 13–33
Kitatani et al., 2020b	8 stroke	OGW	N/A	TA, MG, LG	EMG	TA: swing TA, MG, LG: stance	IMC_Intra_: TA IMC_Inter_: MG, LG	Beta: 15–35

TMW, treadmill walking; OGW, overground walking; N/A, not applicable; TA:, tibialis anterior; MG, medial gastrocnemius; LG, lateral gastrocnemius; BF, biceps femoris; VL, vastus lateralis; VM, vastus medialis; ST, semitendinosus; EEG, electroencephalography; EMG, electromyography; IMC_Intra_, intra-muscular coherence; IMC_Inter_, inter-muscular coherence.

Kitatani et al. (2020a) recruited neurotypical adults, who walked either with tACS or sham- tACS application for 10 min. Each walking condition (stimulation vs. sham) was separated by 5 days. In both walking conditions, one electrode was centered over the left motor cortex at TA’s hotspot, which was the spot with the largest motor response on the right TA using TMS, while the other was centered 3 cm right lateral and upward from the inion (i.e., projecting part at the base of the skull). The frequency of tACS was set individually for each participant by calculating the gait-specific frequency for each participant from 20 stable gait cycles. Therefore, the intensity of tACS was set at 2 mA (range: ±1 mA) at the predetermined frequencies (tACS: 0.872 ± 0.062 Hz; sham: 0.865 ± 0.057 Hz). Right before and after each walking condition, participants underwent neurophysiological assessment during off task (i.e., TMS) and walking (i.e., walking EMG) to capture the effect of each condition. Specifically, participants walked for 5 min on the treadmill while surface EMG from multiple knee and ankle muscles were collected to calculate coherences in the beta band (13–33 Hz) during specific phases of gait. They also underwent TMS testing in which TMS-derived measures were collected for TA and LG to calculate the MEPs from each muscle. From the coherence analyses, results showed that tACS had an effect only on TA IMC_intra_, which showed an increase (post-tACS vs. pre-tACS and post-Sham) during early swing; the rest of the synergistic IMC_Inter_ pairs showed no change. Similarly, only tACS had an effect on TA MEP, which showed increase in amplitude; LG MEP showed no changes in either walking condition. Furthermore, the change in TA MEP was positively correlated to the change of the TA IMC_Intra_; a significant correlation for the LG was not demonstrated.

Findings from this study showed for first time that applying tACS at the individual gait frequency during walking can increase both the beta band coherence and MEP of the TA in neurotypical adults. This effect was only for TA and not for ankle plantarflexors and thigh muscles. Multiple factors may contribute to this finding. First, tACS was applied over TA’s hot spot; therefore, given that the cortical origins of TA were explicitly targeted, it may be expected that the main effect of stimulation would be on TA. Also, it is known that the cortical spot of leg muscles may not be as segregated as in the case of upper extremity muscles, yet the cortical hot spot of leg muscles is also segregated to an extent (Saisanen et al., 2010). Hence, if tACS was applied on the cortical hot spot of different muscles, results may have differed as well. Second, given that only the beta band was reported for all muscles, and ankle plantarflexors and thigh muscles may not have as strong CST connections as the TA, the tACS parameters used in this study may have been optimal for TA but not for the rest of the muscle groups. Although, these findings may have some methodological bias toward TA and no walking behavior was quantified and correlated with neurophysiological measures, this study is very important as it demonstrates that a single session of 10 min of tACS application during treadmill walking may uncover a causal mechanism of the neural control of walking. It also specifically demonstrates the tACS-induced changes in neural control of TA during walking (i.e., IMC_Intra_) and off task (i.e., MEP).

Given these promising results in neurotypical adults, the same group investigated the long-term effect of tACS during walking in chronic stroke patients using a single-blinded crossover study (Kitatani et al., 2020b). Stroke patients walked on treadmill for 10 min either during real tACS and sham-tACS for a total 10 sessions (2 days × 5 weeks). In both walking conditions, the electrode placement was similar to their previous study (Kitatani et al., 2020a); however, tACS was applied over the hot spot of the paretic TA in the ipsilesional hemisphere; therefore, the paretic leg was targeted by the stimulation. Conversely to their previous study, in which tACS frequency was based on individual gait frequency, in this study they applied 1 cycle of current with tACS intensity of 2 mA (i.e., sinusoidal wave: 0–2–0 mA) at the paretic foot contact (frequency range: real tACS—0.50–0.63 Hz; sham—0.57–0.59 Hz). Right before and after each walking condition, participants walked over ground while bilateral surface EMG from TA and both heads of gastrocnemius (MG and LG) were collected [to calculate the TA-TA and LG-MG coherences at the beta band (15–35 Hz) during initial swing (TA-TA) and mid-to-terminal stance (LG-MG)] and underwent over ground walking evaluation. Of the 8 patients, seven completed the pilot study. Given that tACS was applied over the cortical origins of the paretic TA on the ipsilesional hemisphere (i.e., contralateral to the paretic TA), coherences analyses showed significant beta band change due to tACS only for the TA IMC_Intra_ on the paretic side; any other coherence was not significant. For the correlations between coherences and walking behavior, only TA IMC_Intra_ on the paretic side was positively correlated with the change in gait distance; hence the greater the TA IMC_Intra_ on the paretic side was, the greater the distance stroke patients could walk after the intervention.

Findings from this study showed for first time that applying tACS during walking intervention in chronic stroke patients can increase the common neural drive, which is captured by the beta-band intramuscular coherence to the paretic TA, and this increase was associated with reversing a walking deficit (i.e., increasing walking distance). Changes were observed only after tACS and not sham-tACS and on the paretic side. Given that the ipsilesional CST was targeted with tACS and beta band may be a proxy of CST, one could expect changes on the TA but not on the MG and LG. Therefore, the absence of tACS-induced changes in plantarflexors does not necessarily imply that tACS has no potential effect on their common neural drive. Such effects could ensue if tACS was applied over the cortical origins of the paretic gastrocnemius. This remains to be explored. Another promising finding from this study, was the positive association between the paretic TA beta band and the change in walking distance due to tACS. Hence, tACS did not only change the neurophysiology of the paretic TA but also a global walking measure, which can be affected by multiple factors.

The combined findings of both studies added new evidence regarding the role of beta band on the motor descending drive in neurotypical and neurologically impaired walking. In both studies, application of tACS could increase the beta band of TA IMC_Intra_ only and not of any other leg muscles. Given that tACS was applied on the cortical origins of TA and TA IMC_Intra_ was calculated during the swing phase, changes in beta band may reflect tACS-induced changes in CST. This postulation is strengthened by the fact that tACS also increased TA MEP, which reflects changes in CST excitability. Taken together these findings, the beta band oscillations calculated during walking, both neurotypical and neurologically impaired, may be a proxy of CST. Furthermore, both studies pointed out that tACS has the capacity to modulate CST during treadmill walking and subsequently the cortical drive to TA (captured by TA IMC_Intra_). Importantly, these modulations can contribute to the reversal of a walking specific deficit. Finally, no participants in either study reported any adverse events due to tACS; therefore, prolonged tACS application during walking sessions may be safe and feasible. Of course, future work should elaborate more on these methodological aspects (i.e., safety, feasibility, dosage, etc.) (Wu et al., 2021).

Finally, one of the key mechanisms by which tACS is assumed to exert its effect on the brain is entrainment of intrinsic brain oscillations (Vogeti et al., 2022). As we have described, oscillation at different frequencies including the alpha and beta bands is perhaps very important for motor control and walking. However, none of the tACS studies to date has actually used stimulation at the alpha or the beta frequency band. Both studies (Kitatani et al., 2020a,b) targeted the natural gait frequencies, which are much lower than alpha and beta. Thus, the tACS effects observed in these studies cannot be due to the entrainment mechanism. However, they may be due to the second putative mechanism by which tACS exerts its effect, namely, spike-timing-dependent plasticity. More studies are needed to dissect these effects and indeed explore whether entrainment effects when stimulated at brain frequency (alpha and beta ranges) exist and play a functional role.

## Critique

Using CMC and IMC, all studies discussed so far have suggested that the motor descending drive is temporally patterned and exhibits alpha and beta oscillations in neurotypical and neurological impaired walking. Yet, these studies had certain methodological limitations, which should be considered in the interpretation of their findings.

First, the frequency range used to quantify each band varied across studies. For example, Grosse and Brown (2003) reported the 10–20 Hz band to be a proxy of CReST, and since then others used this finding to claim that alpha band is a proxy of CreST. However, based on the conventional classification of frequency ranges, the 10–20 Hz band includes both high alpha and low beta frequencies. It may therefore not accurate to explicitly claim that alpha band is CreST-derived solely from the Grosse and Brown (2003) study. Even within the beta frequency range there is great variability of the actual ranges used. Commonly, the beta band range is 13–30 Hz. However, studies that reported beta band modulation used substantial variations in their definitions of this band (e.g., 13–33 vs. 15–35 Hz). Such variation increases the level of difficulty to synthesize evidence across different studies and to ultimately interpret the underlying mechanisms of each band. An alternative approach could be the reporting of the actual peak frequencies.

Second, not all studies reviewed were consistent in whether arm swing during walking was allowed. This may prove to be an important inconsistency, as recent work, which used coherence analyses, demonstrated that arm swing during walking can modulate lower limb muscle activity *via* both cortical and subcortical pathways (Weersink et al., 2021). Therefore, comparing results from studies in which arm swing varied may be inaccurate. In fact, this is a potential confounding variable, which future studies should control.

## Remaining gaps in knowledge and directions for future research

Despite existing evidence from studies which examined CMC and IMC suggesting a potential frequency specificity (alpha vs. beta band oscillations) of the motor descending drive in neurotypical and neurological impaired walking, certain gaps in knowledge still exist. Future work should address these gaps in order to further elucidate the role of these oscillations in the common neural drive to leg muscles during walking. This in turn will enhance existing and improve the development of new rehabilitation strategies, which focus on locomotor recovery.

While much evidence suggests that beta band oscillations represent a proxy of CST, whether alpha band oscillations are a proxy of CreST is less clear. This discrepancy may be due to several factors. First, the quantification of alpha oscillations may be methodologically more challenging than quantification of beta oscillations. Given the low frequency (i.e., 8–13 Hz) of alpha oscillations, certain methodological parameters in EMG and IMC analysis require additional considerations. Important is the selection of sufficiently long epochs to capture variation at these slower temporal scales, the use of high pass filters at a much lower frequency than what is conventionally used in EMG analysis and the number of epochs, which in turn affects the signal-to-noise ratio (SNR). Also, alpha oscillations may not be as reliably observed as beta or gamma oscillations. Whether this is partly a methodological issue or simply reflects a truly (physiologically speaking) lower SNR remains to be investigated. Furthermore, animal studies showing a more direct relationship between the descending motor tracts and alpha oscillations as observed in EMG and IMC are necessary. Future work in animal models can feasibly quantify whether alpha oscillations observed in hindlimb- EMG and IMC indeed originates from and is driven by CreST. Similarly, using non-invasive techniques, human studies could examine the causal relationships between CreST-derived measures using TMS (i.e., ipsilateral MEPs proxy of CReST) (Ziemann et al., 1999; Taga et al., 2021) off task and coherences of alpha oscillations during walking. If this is indeed the case, then alpha band IMC and CMC can be seen as proxies of CreST activation. Similarly, if indeed alpha oscillations in the cortex affect the descending drive by CreST these may be targeted by tACS therapeutically in stroke recovery to upregulate this pathway and hence facilitate locomotor recovery and walking.

Most of the studies that have investigated alpha and beta oscillations during walking have not quantified biomechanically the walking behavior. Characterizing walking mechanics provides quantitative data on how body, joints, and muscles act during walking. Such evidence can be used to elaborate on the behavioral manifestations of the common neural drive to the leg muscles during walking. Yet, surprisingly, only a few studies reported walking measures in relation to these neural oscillations. In addition, the walking measures employed were global (e.g., walking distance) and hence not specific to the muscles of interest. For example, propulsive impulse during the push off phase and ankle angular velocity during the swing phase can be used as proxies of the plantarflexors and TA, respectively (Charalambous, 2015). Using muscle-specific mechanical measures instead of global measures can present a more direct association with the common neural drive to the muscle of interest. Therefore, future studies should explicitly examine both alpha and beta oscillations in relation to muscle-specific neuromechanical measures in both ankle plantarflexors and dorsiflexors during a muscle group specific gait phase (plantarflexors–stance; dorsiflexors–swing). Such an approach will link the common neural drive to specific neuromechanics of the muscles of interest and can also reveal how this frequency specificity is related to the mechanics of each muscle group that acts around the ankle joint during muscle-specific gait phase.

Furthermore, tACS is a promising NIBS modality which may alter behavior by modulating cortical neural oscillations during walking. Yet, data on possible effects of tACS on walking is very limited, for both neurotypical and neurologically impaired walking. We were only able to identify two studies in the literature, both of which demonstrated a positive effect of tACS on increasing beta band IMCs (both neurotypical adults and chronic stroke patients), CST excitability (neurotypical adults), and walking distance (chronic stroke patients). In both studies, however, tACS targeted solely the cortical origins of TA. Though TA has a crucial role during the swing phase of gait (∼40%), ankle plantarflexors contribute greatly during the stance phase of gait (∼60%) (Winter, 1991). Therefore, future work should focus on targeting the cortical origins of the ankle plantarflexors using tACS during walking and relate the common neural drive to this muscle group with mechanical measures that quantify the function of this muscle group (i.e., propulsive impulse) during the specific gait phase that this muscle is active (i.e., push off) (Neptune et al., 2001). Moreover, it is still unclear which the optimal frequency to be used by tACS is, in order to entrain the intrinsic neural oscillations for walking-specific muscles. While beta oscillations are strong in TA CMC and IMC_Intra_ during the swing phase, it is still unclear whether beta oscillations, alpha oscillations or both are strongly present for the plantarflexors during the stance phase. Therefore, future studies of IMC and CMC should quantify the frequency range of the oscillations present in the muscles of interest during walking. This, in turn can inform the optimal frequency to be used for tACS. Therefore, future work should systematically quantify the tACS parameters used for targeting different walking specific muscles with an end point goal the improvement and/or recovery of walking behavior.

Another remaining question is how these oscillations are altered relative to walking recovery post-stroke. The few studies that examined the neural oscillations during walking in people post-stroke recruited only chronic stroke patients (Nielsen et al., 2008; Kitatani et al., 2016, 2020b). However, being able to track the changes in alpha and beta oscillations in relation to walking behavior across time after stroke onset (acute–subacute–chronic phase) can add to existing evidence of locomotor recovery after stroke. Having knowledge of these recovery mechanisms of post-stroke walking can assist in the improvement of existing and development of new rehabilitation strategies. Future work should track both the neural oscillations and walking mechanics from the acute phase (i.e., 1–7 days after stoke onset) until the chronic phase (i.e., > 6 months after stroke onset) (Bernhardt et al., 2017). Such an approach will indicate how the common neural drive and muscle-specific mechanics can contribute to walking recovery after stroke. Findings from such studies will inform theories of locomotor recovery after stroke.

## Methodological considerations

Collecting neurophysiological signals using either EEG and/or EMG and calculating neural oscillations during walking is potentially feasible in both neurotypical adults and neurological impaired patients (e.g., stroke). However, both neurophysiological techniques have inherently certain methodological limitations, which should be considered. Next, we will present and discuss the methodological considerations related to collection and analysis of EEG and EMG signals.

The two main considerations for using EEG are the contamination of the signal by artifacts and the estimation of the true sources of oscillations in the brain from surface recordings measured from the scalp. While there also exist artifacts of technical nature, perhaps the most challenging artifacts in scalp EEG are of biological nature, namely, contamination by eye movements, by cardiac activity and in addition movement artifacts and EMG contamination produced by face and scalp muscles. While these artifacts can be quite large, there are now several approaches which can lead to relatively successful artifact handing (Kaya, 2019) and artifact rejection (Kanoga and Mitsukura, 2017). Identifying the true source of the oscillations from surface EEG recordings is a considerable challenge known as the inverse problem. While there exist no unique solutions to this inverse problem, several methods, each one making assumptions about the underlying sources, have been meanwhile validated (Hillebrand et al., 2005). Some of these techniques also provide simultaneous artifact rejection in addition to source localization and source reconstruction (Brookes et al., 2008). However, in the setting of scalp EEG with only a few leads, which is likely to be the setting of recording during walking in stroke patients, one may not be able to necessarily perform such source reconstruction, as this typically requires the anatomical MRI of the patient and in addition co-registration of the electrode positions with respect to the MRI. The same considerations also apply to other clinical settings. In such cases one can still work with surface recordings directly as obtained on the scalp. In this setting two major problems confound the inference. The first problem is the “non-silent” (i.e., non-zero) reference channel that introduces contamination of the local signals by signals measured elsewhere. The second problem is volume conduction, which is the contamination of signal from elsewhere propagating through the tissue (Nunez and Srinivasan, 2006). In previous work, we have assessed the impact of different reference electrodes (different montages) and different signal measures (Anastasiadou et al., 2019). The “safest” choice in obtaining a relatively local activity in the clinical setting with a limited number of electrodes may be the bipolar montage (Anastasiadou et al., 2019). This provides an estimate of the local signal between the two electrodes in the bipolar pair. In this way the issue of reference electrode (signal is difference between two “active” electrodes) is addressed explicitly and also the influence of volume conduction is mitigated somewhat in that that far sources mutually cancel out (Nunez and Srinivasan, 2006). Therefore, the bipolar montage may be a practical compromise solution in the setting of assessing cortical/corticospinal activity during walking in stroke patients.

As with EEG, the collection, extraction, and analysis of EMG signal should be conducted bearing in mind a few considerations (Farina et al., 2014). These considerations can be divided into two groups. The first is related to physiological properties of the muscle(s) of interest (e.g., spatial distribution and conduction velocities of the muscle fibers, thicknesses of subcutaneous fat tissue over the target muscle, etc.) (De Luca, 1997; Farina et al., 2002). Given that dorsiflexors and plantarflexors are the two main muscle groups of interest in studies that examined neural oscillations during walking, it is crucial to bear in mind that intra- and inter-muscle group differences exist in muscle physiology (e.g., dorsiflexors are more susceptible to the fatigue than plantaflexors) (Belanger and McComas, 1985) and neural control (i.e., common neural drive may differ within plantarflexors) (Hug et al., 2021). For example, prolonged testing of walking may fatigue dorsiflexors faster than the plantarflexors; hence, the antagonistic coupling between these two muscle groups may be affected. Therefore, small bouts of walking trials may be more appropriate and feasible, especially in clinical cohorts. The second set of considerations is about the methodology used to collect EMG. Factors such as the preparation and placement of electrode over the muscle(s) of interest play a role (Roy et al., 1986). To control this consideration, standardized guidelines for this matter have been established (Hermens et al., 1999).

## Conclusion

Quantifying neural oscillations and neural-driven muscle oscillations during walking is a fascinating approach to characterize the neural control of walking “online.” Here we focused specifically on the putative differential role of alpha and beta oscillations in the temporal patterning of the motor descending drive in neurotypical and neurologically impaired walking. Conjecturing that each of these oscillations may be a proxy of one of the two major motor descending pathways, one could study the characteristics of each of the two oscillations relative to walking in an attempt to elucidate the function of each tract during walking. While there is evidence that beta oscillations in IMC_Intra_ and CMC could be viewed as a proxy of CST descending neural drive, whether alpha oscillations in the respective signals can be viewed as a proxy of CreST remains unclear. Here we have identified gaps in the literature, which when addressed could help answer this question. Furthermore, we have shown that, at least intrinsic beta-oscillations, can be modulated using tACS. These modulations also modified CST excitability and walking behavior. We conclude that the careful characterization of frequency-specific oscillations in electrical neural and muscular signals may represent a valuable path toward a better understanding of the neural control of walking.

## Author contributions

CCC wrote the draft manuscript. Both authors conceptualized the review, contributed to manuscript revision, read, and approved the submitted version.
